# Clinic, neuropathology and molecular genetics of frontotemporal dementia: a mini-review

**DOI:** 10.1186/2047-9158-2-8

**Published:** 2013-04-19

**Authors:** Xiao-dong Pan, Xiao-chun Chen

**Affiliations:** 1 Department of Neurology, Union Hospital of Fujian Medical University, 29 Xinquan Road, Fuzhou 350001, China; 2 Key Laboratory of Brain Aging and Neurodegenerative Disease; Fujian Institute of Geriatrics, Union Hospital of Fujian Medical University, 29 Xinquan, Road, Fuzhou 350001, China; 3 Centre of Neurobiology, Fujian Medical University, 88 Jiaotong Road, Fuzhou, 350001, China

**Keywords:** bvFTD, Nonfluent/agrammatic variant, Semantic variant, Logopenic variant, Molecular genetics, *MAPT*, *GRN*, *C9ORF72*

## Abstract

Frontotemporal lobar degeneration (FTLD) represents a group of clinically, neuropathologically and genetically heterogeneous disorders with plenty of overlaps between the neurodegenerative mechanism and the clinical phenotype. FTLD is pathologically characterized by the frontal and temporal lobar atrophy. Frontotemporal dementia (FTD) clinically presents with abnormalities of behavior and personality and language impairments variants. The clinical spectrum of FTD encompasses distinct canonical syndromes: behavioural variant of FTD (bvFTD) and primary progressive aphasia. The later includes nonfluent/agrammatic variant PPA (nfvPPA or PNFA), semantic variant PPA (svPPA or SD) and logopenic variant PPA (lvPPA). In addition, there is also overlap of FTD with motor neuron disease (FTD-MND or FTD-ALS), as well as the parkinsonian syndromes, progressive supranuclear palsy (PSP) and corticobasal syndrome (CBS). The FTLD spectrum disorders are based upon the predominant neuropathological proteins (containing inclusions of hyperphosphorylated tau or ubiquitin protein, e.g transactive response (TAR) DNA-binding protein 43 kDa (TDP-43) and fusedin-sarcoma protein in neurons and glial cells) into three main categories: (1) microtubule-associated protein tau (FTLD-Tau); (2) TAR DNA-binding protein-43 (FTLD-TDP); and (3) fused in sarcoma protein (FTLD-FUS). There are five main genes mutations leading clinical and pathological variants in FTLD that identified by molecular genetic studies, which are chromosome 9 open reading frame 72 (*C9ORF72*) gene, granulin (*GRN*) gene, microtubule associated protein tau gene (*MAPT*), the gene encoding valosin-containing protein (*VCP*) and the charged multivesicular body protein 2B (*CHMP2B*). In this review, recent advances on the different clinic variants, neuroimaging, genetics, pathological subtypes and clinicopathological associations of FTD will be discussed.

## Introduction

Frontotemporal dementia (FTD) represents a group of clinically, neuropathologically and genetically heterogeneous disorders. It is a range of progressive dementia syndromes associated with focal atrophy of the orbitomesial frontal and anterior temporal lobes. And the term frontotemporal lobar degeneration (FTLD) to describe the pathological syndrome.

In 1892, Arnold Pick described a patient with progressive aphasia and lobar atrophy [1]. In 1911, Alois Alzheimer described the histopathological status related to these patients, pointing the absence of senile plaques and neurofibrillary tangles, and the presence of argyrophilic neuronal inclusions (later called “Pick bodies”) and swollen cells (later called “Pick cells”) at neuropathological examination [2]. However, during the 20th century, these patients with frontotemporal lobar degeneration were generically referred to as patients with dementia, being often diagnosed with Alzheimer disease (AD) [2]. In 1994, two major research groups from Lund and Manchester proposed clinical and neuropathological criteria for the diagnosis of FTD [3]. In 1998, Neary et al [4] reported a consensus on clinical diagnostic criteria of frontotemporal lobar degeneration in the American Academy of Neurology (AAN).

FTD often begins when the patient is in the fifth to seventh decades. Epidemiological studies suggest that FTD is the second most common cause of dementia in individuals younger than 65 and is just less common than Alzheimer’s disease (AD) within this age group [2,5]. Of over 65 years old dementia, the incidence of FTD ranks fourth, the top three is Alzheimer’s disease, Lewy body dementia and vascular dementia [5-7]. There do not appear to be any clear gender differences in susceptibility [8-11]. Among the FTD clinical syndromes, sex distribution appears to vary from one subtype to the next. Several studies report a male predominance in bvFTD and SD, and a female predominance in PNFA [5,12]. There is a wide range in durations of illness (2-20 years) partly reflecting different underlying pathologies.

In the past few years, significant advances have been seen in the etiology, pathogenesis, and genetics, pathology and clinical phenotype of FTD, and many new terminology and classification of FTD have emerged. In this review, recent advances on the different clinical variant phenotypes, neuroimaging, hereditary forms, pathological subtypes and clinicopathological associations of FTD will be discussed.

## FTLD clinical presentation: classification and characteristics

Frontotemporal lobar degeneration (FTLD) includes a group of progressive degenerative disorders characterised by progressive behavioural change, executive dysfunction and language difficulties [13]. Unlike AD, behavioral symptoms predominate in the early stages of FTLD. The clinical heterogeneity in familial and sporadic forms of FTD is remarkable, with patients demonstrating variable mixtures of disinhibition, dementia, PSP, CBD, and motor neuron disease [13,14]. Early symptoms are divided among cognitive, behavioral, and sometimes motor abnormalities, reflecting degeneration of the anterior frontal and temporal regions, basal ganglia, and motor neurons [13,14]. FTLD is a powerful model to study emotion, social cognition and language organization in the brain.

## Behavioral variant frontotemporal dementia (bvFTD)

bvFTD has the highest prevalence amongst the FTD clinical syndromes, accounting for approximately 70% of all FTD cases [2,5]. The onset of bvFTD is typically before the age of 65 years, with an average onset age of 58 years [12,15]. Patients with this clinical variant present with marked changes in personality and behaviour and with relative preservation of the cognitive functions praxis, gnosia and memory [13,16]. Common behavioral deficits include apathy, disinhibition, weight gain, food fetishes, compulsions, euphoria, and an absence of insight into their condition. Poor business decisions and difficulty organizing work tasks are common. Cognitive testing typically reveals spared memory but impaired executive functions [3,13,17]. bvFTD has been associated with symmetrical ventromedial frontal, orbital frontal, and insular atrophy and left anterior cingulate atrophy [18]. Behavioural assessment is at the core of assessment in patients with potential bvFTD and seems to be more sensitive in distinguishing bvFTD from AD than standard cognitive testing. The ‘International Behavioral Variant FTD Criteria Consortium’ developed international consensus criteria for bvFTD. Subclassifications were made in possible bvFTD defined by clinical criteria, probable bvFTD supported by neuroimaging data, and definite bvFTD confirmation by neuropathological evidence or a pathogenic mutation [17].

### Primary progressive aphasia (PPA)

PPA is a language disorder that involves changes in the ability to speak, read, write and understand what others are saying. It is associated with early temporal lobe atrophy or brain lesions around the lateral fissure. PPA represents a spectrum of selective language disorders and the variant of PPA might provide clues to the underlying pathology [19]. Currently cerebrospinal fluid (CSF) biomarkers have limited ability to identify PPA reliably, which might be explained by the pathological heterogeneity. An indication of lower Aβ1-40 levels or the lower T-tau to Aβ1-42 ratio in FTD, might be useful to distinguish patients from subjects AD and control subjects [20,21]. In 2011, criteria were adopted for the classification of PPA into three clinical subtypes: nonfluent/agrammatic variant PPA (nfvPPA or PNFA), semantic variant PPA (svPPA or SD) and logopenic variant PPA (lvPPA) [22]. The proposal is an important step forward in establishing the consistency of terminology and classification of PPA. The diagnostic recommendations include two steps. First, the patient should meet the basic standard of PPA, which is an initial presentation of significant damage to language and accompanied by limited daily living skills and other cognitive impairment. Second, we need to assess the main language domains, including verbal generation, repeat, and understanding of words and syntax, naming, semantic knowledge and reading/spelling characteristics. Finally, clinical variants will be divided basing on specific speech and language features characteristic of each subtype. Classification can then be further specified as “imaging-supported” if the expected pattern of atrophy is found and “with definite pathology” if pathologic or genetic data are available. Therefore, the new proposal has a clear practical significance.

nfvPPA or PNFA is the second most prevalent presentation of FTLD, accounting for a large 25% [12]. The feature of nfvPPA includes grammatical error making agrammatism and/or laborious speech, but relatively preserved language comprehension. Apraxia of speech (AOS) or orofacial apraxia is frequently accompanying the aphasia [15]. Semantic variant PPA (svPPA) or SD presents in 20–25% of the FTLD patients [12]. svPPA presents a significantly impaired naming ability and word comprehension, while speech production is spared [22,23]. LvPPA or logopenic progressive aphasia [24] is marked by impaired word finding and difficult repetition. lvPPA showes an impaired ability to repeat sentences or phrases, spontaneous speech and a single word extracting when naming. Unlike other FTD subtypes, lvPPA generally does not produce changes in behavior or personality until later stages of the disease. Most people with progressive aphasia maintain the ability to care for themselves, keep up outside interests and, in some instances, remain employed for a few years after onset of the disorder. LPA is mostly associated with a neuropathological diagnosis of AD [25]. Clinical distinction between bvFTD, nfvPPA and svPPA is often complicated for that overlap of the clinical syndromes between them can occur in advanced stages of disease.

### FTLD overlaps with other clinical syndrome

The subcortical nuclei and motor systems are involves in FTLD variants. Motor neuron dysfunction (MND) has been described in 40% of the FTLD patients, referred to as FTLD-MND. The common comorbidity of Amyotrophic lateral sclerosis (ALS) with behavior abnormality, cognitive impairment or dementia has been noticed [26,27]. FTLD may precede, follow or coincide with the onset of motor symptoms [28]. Other disorders are closely related to FTLD, including progressive supranuclear palsy (PSP) syndromes, corticobasal syndrome (CBS), FTD with parkinsonism (FTDP) and argyrophilic grain disease (AGD). PSP and CBS are two common atypical parkinsonian syndromes demonstrating cognitive and behaviour disorders that overlap with FTD. Of them, behavioral and cognitive dysfunction promotes the development of dementia. And extrapyramidal system symptoms present as bradykinesia, abnormal posture, rigidity, but tremor uncommon. The co-occurrence of AGD in ALS is not uncommon, and comparable with that in a number of diseases belonging to the tauopathies or α-synucleinopathies [29]. Motion system damage presents as muscle atrophy. FTDP-17 showing genetic linkage to chromosome 17, presents with a clinical syndrome of autosomal dominant disinhibition, dementia, parkinsonism, and amyotrophy [30]. The clinical picture resembles bvFTD, while cognitive deficits include anterograde memory dysfunction in an early stage, later the deterioration of visuospatial function, orientation and global memory are gradually presented. Motor signs typically include the symmetrical bradykinesia without resting tremor, in combination with axial rigidity and postural instability. The early clinical presentation of AGD is similar to AD but it is less aggressive, with patients maintain mild cognitive impairment (MCI) for many years [31].

## Neuroimaging in FTLD

Neuroimaging plays a critical role in diagnosis of FTD. Neuroimaging investigations can reliably differentiate FTLD subtypes from other dementias, and can help clinical diagnostics based on neuropsychiatric symptoms. Different clinical variatents have different forms of brain atrophy, which can be used for some important clues for early clinical differential diagnosis. DTI can detect white matter damage, different subtypes of white matter injury also help clinical early differential diagnosis of clinical subtypes of FTLD [32]. Functional neuroimaging techniques, such as [^99^mTc]-hexamethylpropyleneamine oxime single-photon emission computed tomography (SPECT) [33] or [^18^ F]-fluorodeoxyglucose (FDG)-PET are increasingly being used to help with the diagnosis of FTLD [34]. Arterial spin labeling imaging (ASL) like FDG-PET can be used for showing the hypometabolism in brain regions. Hypometabolism on FDG-PET is detected consistently and reliably in frontal brain regions in patients with bvFTD compared with those with AD, who show posterior cingulate hypometabolism early in the disease process [34]. However, in patients showing clear brain atrophy on structural MRI, little additional diagnostic benefit is gained by doing a PET scan, because focal atrophy is a positive predictive marker of FTD. The imaging presentation of nfvPPA indicates that predominant left posterior fronto-insular atrophy on MRI or predominant left posterior fronto-insular hypoperfusion or hypometabolism on SPECT or PET. Imaging-supported svPPA show predominant anterior temporal lobe atrophy and/or these brain regions hypoperfusion or hypometabolism on SPECT or PET. The imaging of lvPPA must show at least one of predominant left posterior perisylvian or parietal atrophy on MRI and/or hypoperfusion or hypometabolism on SPECT or PET [22,25,35-37].

Future in FTLD are very similar to imaging in AD–early detection, molecular diagnosis, monitor disease progression and validate and implement disease modifying therapies. Specific anatomic patterns in FTLD, and an amyloid-PET neuroimaging, which uses the amyloid-β-detecting ^11^C-Pittsburgh compound B, has shown promising results in discriminating or rule-out atypical AD and FTD cases [38,39] particularly those presenting with language deficits rather than behavioural changes. Micro PET with ^18^ F-THK523 in Tau transgenic mice have been reported [40]. Tau specific imaging scanner that uses the ^18^ F-THK523 may be a promising technique coming soon in future [40].

## FTLD Neuropathology and cliniconeuropathological correlations

### Neuropathology

The subtypes of underlying pathological changes in patients with FTD are classified on the basis of the pattern of protein deposition, and are referred to collectively as frontotemporal lobar degeneration. Under a microscope, common pathologic changes of FTLD are atrophy brain region presenting neuronal loss, spongy change and gliosis in cortices of atrophied frontal and temporal lobes [41,42].

FTD have identified novel genetic defects and a chromosomal locus in hereditary forms of FTLD, as well as novel clinicneuropathological associations. Immunohistochemistry allows subcategorization of these disorders into specific proteinopathies based on the major constituent of the inclusions.

(1) **FTLD-tau**: One subtype is presented as neurons  and glial cells containing inclusions of  hyperphosphorylated tau protein, referred to as  FTLD-tau [43]. Tau pathology was associated with  FTD with parkinsonism, PSP syndromes [44], CBS  [45] and AGD [31].

(2) **FTLD-Ubiquitin or FTLD-U**: Over 50% of the  FTLD patients presented with tau-negative  ubiquitin staining inclusions, referred to as FTLD- Ubiquitin or FTLD-U [46]. In 80–95% of this  group, inclusions were found to be composed of  transactive response (TAR) DNA-binding protein  43 kDa (TDP-43) [47-49], referred to as FTLD-TDP  [50], or TDP-43-negative FTLD-U cases having  inclusions of fusedin-sarcoma protein (FUS), referred  to as FTLD-FUS [43,49,51,52]. However, in a small  number of FTLD-U patients, the inclusion protein  remains unclear. This group is referred to as FTLD- ubiquitin proteasome system (FTLD-UPS) [46,53].

(3) Dementia lacking distinctive histopathology  (DLDH) [54,55].

(4) Other rare types: dementia with basophilic  inclusion body, neuronal intermediate filament  inclusion disease [52].

### Association between pathology and clinical phenotype

Associations were noted between the clinical FTLD subtypes and underlying proteinopathies, but a strict one to one relationship is lacking. bvFTD is mostly associated with FTLD-TDP43, some cases are also correlated with FTLD-tau (PiD subtype). An FTLD-FUS proteinopathy is invariantly associated with a clinical diagnosis of bvFTD, either with or without the signs of MND. Microscopic assessment of nfaPPA at autopsy often reveals FTLD associated with a tauopathy (FTLD-tau). nfaPPA is commonly associated with tau pathology, especially when AOS or orofacial apraxia is present. In most cases, the pathology underlying nfaPPA was mixed, including FTLD-tau and FTLD-TDP43, but with a predominance of FTLD-tau pathology. SvPPA is, in most cases, linked with TDP-43-immunoreactive pathology, although tau pathology is sometimes observed. Some patients with predominantly right temporal lobe atrophy (RTLA) are usually diagnosed clinically with either bvFTD or svPPA [56]. It has been suggested that patients with bvFTD and RTLA have FTLD-tau pathology, whereas patients with svPPA and RTLA have FTLD-TDP43 pathology [56]. svPPA patients who often present with acalculia is associated with FTLD-tau [57]. LvPA is predominantly associated with AD pathology. Many patients with lvPPA often have underlying AD pathology [58-60]. CSF tau:amyloid-β ratio [37] and PiB PET imaging [25] studies can be helpful in the identification of patients who are more likely to have lvPPA than nfaPPA. A recent study showed that 93% of patients with lvPPA on amyloid PET imaging was found PiB positive, but svPPA only 9%, nfvPPA is 13%, which suggests that there is some common pathological features between lvPPA and AD [59].

Tau pathology was also associated with FTLD with parkinsonism, PSP, CBD, AGD and Pick bodies (PiD) [30,44,61]. Similarly, TDP-43 and FUS proteinopathies are also commonly found in MND with or without FTLD [52]. Although these are relatively strong associations, they are not absolute, and it is currently not possible to predict with certainty the underlying pathology of specific FTLD syndromes.

Extrapyramidal system symptoms accompanied with apraxia indicates the presence of CBS, which mostly attributes to Tau disease. While dementia with FTLD-MND syndrome mostly attributes to TDP-43 pathology [15,45,57]. These suggest that it is contributing to therapeutic strategy for FTLD through elucidating the relationship between pathological and clinical presentation.

## Molecular genetics

Approximately 40% of patients with FTD have a family history [8]. bvFTD is the most prominent subtype with family history, especially when concomitant symptoms of MND are present (60%), while SvPPA appeared to be the least hereditary FTLD subtype (<20%) [62]. Molecular genetic studies have identified several common (*MAPT, GRN*) and rare (*VCP, CHMP2B, TARDBP, FUS*) genetic factors in hereditary FTLD over recent years. In 1998 microtubule associated protein tau (MAPT) gene was found, which located on chromosome 17q21.32, in which 13% of the cases is FTDP-17, those who have parkinson-like symptoms [63]. In 2004, the gene encoding valosin-containing protein (VCP) gene was found, located on chromosome 9p13.3, which is associated with the genes that cause FTLD in association with inclusion body myopathy and Paget’s disease [64,65]. In 2005, the charged multivesicular body protein 2B (CHMP2B, also known as chromatin-modifying protein 2B) gene mutations, located on chromosome 3p11.2, was discovered in a large Danish cohort with familial FTLD [66,67]. In 2006, a mutated gene located on chromosome 17q21.31 are identified as the granule protein precursor (PGRN), only 1.7 M nucleotide away from the MAPT [68,69]. Approximately 10% of patients with FTLD is associated with this gene. In 2011, chromosome 9 open reading frame 72 (C9ORF72) gene was newly identified in FTLD [70,71].

Overall, patients with mutations in *GRN, MAPT* and *C9ORF72* together account for at least 17% of total FTD cases [53,72]. Summed *C9ORF72, GRN* and *MAPT* mutation frequencies were 32-40% [70,73]. Mutations in *VCP* and *CHMP2B* are rare, each explaining less than 1% of the familial FTLD.

The MAPT gene mutations often lead to FTLD-Tau pathological changes. The Both *PGRN* and *C9ORF72* mutations can cause FTLD-TDP-43. The symmetry frontal lobe atrophy in bvFTD patients is associated with *C9ORF72* and *MAPT* gene mutations, whereas the asymmetry of frontal lobe atrophy in bvFTD patients is associated with *PGRN* gene mutations [74]. The large hexanucleotide repeat expansion located within the non-coding portion of *C9ORF72* is the cause of chromosome 9-linked ALS and FTLD [70]. These suggest that specific gene mutations may affect the patterns of neuroanatomic injury in the development of frontal lobar atrophy.

*C9ORF72* is expressed as three major transcripts and the expanded G4C2 repeat is located in the proximal regulatory region of *C9ORF72*[70,73], upstream of one and in the first intron of the two other transcripts. Repeat expansion results in near complete loss of expression of the major gene transcripts. The pathogenic expansion was non-penetrant in individuals younger than 35 years, 50% penetrant by 58 years, and almost fully penetrant by 80 years. In normal population, the size of the G4C2 repeat ranges from 3 to 25 units, which is expanded to at least 60 units in FTLD patients [70,71,73].

The clinical phenotype of *C9ORF72* mutation often presents as bvFTD or ALS or FTD co-morbidities ALS. In patients with bvFTD, *C9ORF72* mutation is more common than *MAPT* or *PGRN*. Anxiety or agitation, and memory dysfunction (often episodic memory) is a common clinical feature. Those cases underlines that the hexanucleotide repeat expansion in chromosome 9 could be also associated with early onset psychiatric presentations [75]. Overexpressed p-62 inclusion body lesions in the cerebellum is one of pathological features [76]. Neuroimaging presents as the symmetrical frontal and/or temporal lobe atrophy, and parietal, occipital and cerebellar atrophy can also appear. Therefore, *C9ORF72* mutation is not only associated with cortical anatomic site but also with subcortical structures. However, it is unclear for the exact mechanism of action of this “repeat amplification *C9ORF72* gene”.

The clinical and pathological heterogeneity in FTLD causes a challenge for diagnosis of FTLD. A helpful supplement for clinical evaluation using genetics and biological markers testing can significantly improve the forecast of potential histopathology *in vivo*. We summarize clinical phenotypes, molecular pathological and genetic spectrum in FTLD in Table 1. And the profile of main genes mutations and its possible disease mechanisms in FTLD is shown in Table 2.

**Table 1:** **Clinical phenotypes, pathological and molecular genetic spectrum in FTLD **[13,19,22,35,72,77,78]

**Core clinical feature**	**Praxiological obstacle**	**Logopathy**			**Extrapyramidal symptoms + praxiological obstacle**		**Dyskinesia + praxiological obstacle**
**Clinical syndrome**	Behavioral variant Frontotemporal dementia (bvFTD)	Primary progressive aphasia(PPA)			Corticobasal degeneration (CBD)	progressive supranuclear palsy (PSP)	FTD-MND/ALS
		nonfluent/agrammatic variant PPA (nfvPPA)	Semantics variation (svPPA)	logopenic variant PPA (lvPPA)			
**Brain morphologically affected parts**	Prefrontal lobe and temporal lobe	Left posterior frontal lobe, insula	The front/ventral temporal lobe	left posterior superior temporal lobe and medial parietal lobe	Frontal and temporal lobe, Basal ganglia	Basal ganglia and brainstem	Cortex and motor neuron
**Biochemistry and Neuropathology**	FTLD-Tau (Pick type, 3R-tau) FTLD-TDP43	FTLD-Tau more than FTLD-TDP43, AD like pathological visible	Most belongs to FTLD-TDP43; AD like pathological rare	AD-like pathological common; FTLD-TDP43 visible	FTLD-Tau (CBD type,4R-tau) common	FTLD-Tau (PSP type, 4R-tau) common	FTLD-TDP43, FTLD-FUS
**Causative and Associated Genes**	*C9ORF72*	*PGRN,*	*C9ORF72*		*PGRN*	*MAPT*	*C9ORF72*
	*PGRN*	*C9ORF72*	*PGRN*		*MAPT*	*PGRN*	*FUS*
	*MAPT*	*MAPT*	*MAPT*	*PGRN*	*C9ORF72*	*C9ORF72*	*VCP*
	*VCP*	*VCP*	*VCP*		*VCP*	*VCP*	
	*CHMP2B*	*CHMP2B*	*CHMP2B*		*CHMP2B*	*CHMP2B*	

Note: 3R tau: three microtubulebinding repeats; 4R tau:four microtubule-binding repeats.

**Table 2:** The profile of main genes mutation and its possible disease mechanisms in FTLD

**Gene symbol**	***MAPT***	***C9ORF72***	***PGRN***	***VCP***	***CHMP2B***
**Full name**	**Microtubule -associated protein tau**	**Chromosome 9 open reading frame 72**	**Progranulin**	**Valosin containing protein**	**Chromatin modifying protein 2B**
**Chromosomal localization**	**17q21.32**	**9p21.2**	**17q21.32**	**9p13.3**	**3p11.2**
*.*	*● MAPT *gives rise to six isoforms: three isoforms containing three amino-acid repeats (3R), and three isoforms with four repeats (4R) [79].	*● *expressed as three major transcripts, the expanded G4C2 repeat is located in the proximal regulatory region of *C9ORF72*[70,73].	*● *encodes progranulin, a ubiquitously expressed growth factor precursor consisting of 7.5 granulin peptides.	*● *Encodes a ubiquitously expressed member of a family of ATPases associated with a wide range of cellular functions [85].	*● *Encodes a component of the heteromeric ESCRT-III complex with functions in the endosomal-lysosomal and the autophagic protein degradation pathway.
**Functions and possible role in the disease mechanism**	*●* Mutations result in a change in ratio of 3R to 4R tau isoforms. Mutations affect the normal function of the tau protein to stabilise microtubules, increase the tendency of tau to form neurotoxic aggregates and disturb neuronal plasticity and axonal transport [80].	*● *Repeat expansion results in near complete loss of the major gene transcripts. And accumulation of transcripts harboring the expanded G4C2 repeat in nuclear RNA foci [70].	*● *a wide range of biological processes such as inflammation and wound repair, or in pathological conditions including tumorigenesis [82].	*● *mutations reside at the interface between the D1 ATPase and the N-domain of the CDC48-like protein [85].	*● *Expressed in neurons of all major brain regions. It is critical for development, sexual differentiation [88] and neuronal survival [89].
		*● *G4C2 repeat leads to neuronal cytoplasmic inclusions throughout theentire cortical thickness [81].	*● *Neurotrophic function involved in neuronal survival and neurite outgrowth [83,84].	*● *mutations disturb ubiquitin-proteasome mediated protein degradation, autophagy, or both [86,87].	*● *Mutations affect the C-terminal end of the protein due to aberrant splicing [78].
		*● *unidentified mechanisms exist.			
**Estimated mutation frequency [**[69,72,79,81,90]	0-50%	14-48%	3–26%	<1%	<1%

## Conclusion

In this review, we have systematically updated current understanding on clinical, genetic, neuropathological, and neuroimaging perspectives of FTD. This review aims to arouse awareness of FTD among clinicians, in particular when the overlapping clinical and neuroimaging characteristic features among dementia and different subtypes of FTD remain great challenges for clinicians. Identification of biomarkers for clinical diagnosis and differentiation FTD from other dementia is warranted for future studies. Gene diagnosis test should be considered for familial cases suspected with FTD in clinic practice. Longitudinal clinicopathological correlation studies will further elucidate the network functions of human brain in this complex disorder with behaviour, speech and motor abnormality. With further understanding of the genetics, clinical and neuropathological basis of FTD, we can visualise that FTD will be further clinically defined. And new consensus will be developed for better guidance in clinical practice.

## Competing interests

The authors declare that they have no competing interests.

## Authors’ contributions

XdP collected the reference materials and drafted the manuscript. XcC revised the manuscript. All authors read and approved the final manuscript.

## References

[B1] Rossor MN Pick’s disease: a clinical overview Neurology 2001 56 S3 S5 10.1212/WNL.56.suppl_4.S3 11402142

[B2] Snowden JS Neary D Mann DM Frontotemporal dementia Br J Psychiatry 2002 180 140 143 10.1192/bjp.180.2.140 11823324

[B3] The Lund and Manchester Groups Clinical and neuropathological criteria for frontotemporal dementia J Neurol Neurosurg Psychiatry 1994 57 416 418 8163988 10.1136/jnnp.57.4.416 PMC1072868

[B4] Neary D Snowden JS Gustafson L Passant U Stuss D Black S Freedman M Kertesz A Robert PH Albert M Frontotemporal lobar degeneration: a consensus on clinical diagnostic criteria Neurology 1998 51 1546 1554 10.1212/WNL.51.6.1546 9855500

[B5] Ratnavalli E Brayne C Dawson K Hodges JR The prevalence of frontotemporal dementia Neurology 2002 58 1615 1621 10.1212/WNL.58.11.1615 12058088

[B6] Arvanitakis Z Update on frontotemporal dementia Neurologist 2010 16 16 22 10.1097/NRL.0b013e3181b1d5c6 20065792 PMC3632348

[B7] Barker WW Luis CA Kashuba A Luis M Harwood DG Loewenstein D Waters C Jimison P Shepherd E Sevush S Relative frequencies of Alzheimer disease, Lewy body, vascular and frontotemporal dementia, and hippocampal sclerosis in the State of Florida Brain Bank Alzheimer Dis Assoc Disord 2002 16 203 212 10.1097/00002093-200210000-00001 12468894

[B8] Rosso SM Donker Kaat L Baks T Joosse M de Koning I Pijnenburg Y de Jong D Dooijes D Kamphorst W Ravid R Frontotemporal dementia in The Netherlands: patient characteristics and prevalence estimates from a population-based study Brain 2003 126 2016 2022 10.1093/brain/awg204 12876142

[B9] Mercy L Hodges JR Dawson K Barker RA Brayne C Incidence of early-onset dementias in Cambridgeshire, United Kingdom Neurology 2008 71 1496 1499 10.1212/01.wnl.0000334277.16896.fa 18981371

[B10] Ikeda M Ishikawa T Tanabe H Epidemiology of frontotemporal lobar degeneration Dement Geriatr Cogn Disord 2004 17 265 268 10.1159/000077151 15178933

[B11] Hou CE Yaffe K Perez-Stable EJ Miller BL Frequency of dementia etiologies in four ethnic groups Dement Geriatr Cogn Disord 2006 22 42 47 10.1159/000093217 16682792

[B12] Johnson JK Diehl J Mendez MF Neuhaus J Shapira JS Forman M Chute DJ Roberson ED Pace-Savitsky C Neumann M Frontotemporal lobar degeneration: demographic characteristics of 353 patients Arch Neurol 2005 62 925 930 10.1001/archneur.62.6.925 15956163

[B13] Piguet O Hornberger M Mioshi E Hodges JR Behavioural-variant frontotemporal dementia: diagnosis, clinical staging, and management Lancet Neurol 2011 10 162 172 10.1016/S1474-4422(10)70299-4 21147039

[B14] Lillo P Garcin B Hornberger M Bak TH Hodges JR Neurobehavioral features in frontotemporal dementia with amyotrophic lateral sclerosis Arch Neurol 2010 67 826 830 10.1001/archneurol.2010.146 20625088

[B15] Josephs KA Hodges JR Snowden JS Mackenzie IR Neumann M Mann DM Dickson DW Neuropathological background of phenotypical variability in frontotemporal dementia Acta Neuropathol 2011 122 137 153 10.1007/s00401-011-0839-6 21614463 PMC3232515

[B16] Bathgate D Snowden JS Varma A Blackshaw A Neary D Behaviour in frontotemporal dementia, Alzheimer’s disease and vascular dementia Acta Neurol Scand 2001 103 367 378 10.1034/j.1600-0404.2001.2000236.x 11421849

[B17] Rascovsky K Hodges JR Knopman D Mendez MF Kramer JH Neuhaus J van Swieten JC Seelaar H Dopper EG Onyike CU Sensitivity of revised diagnostic criteria for the behavioural variant of frontotemporal dementia Brain 2011 134 2456 2477 10.1093/brain/awr179 21810890 PMC3170532

[B18] Rohrer JD Lashley T Schott JM Warren JE Mead S Isaacs AM Beck J Hardy J de Silva R Warrington E Clinical and neuroanatomical signatures of tissue pathology in frontotemporal lobar degeneration Brain 2011 134 2565 2581 10.1093/brain/awr198 21908872 PMC3170537

[B19] Grossman M Primary progressive aphasia: clinicopathological correlations Nat Rev Neurol 2010 6 88 97 10.1038/nrneurol.2009.216 20139998 PMC3637977

[B20] Irwin DJ McMillan CT Toledo JB Arnold SE Shaw LM Wang LS Van Deerlin V Lee VM Trojanowski JQ Grossman M Comparison of cerebrospinal fluid levels of tau and Abeta 1-42 in Alzheimer disease and frontotemporal degeneration using 2 analytical platforms Arch Neurol 2012 69 1018 1025 10.1001/archneurol.2012.26 22490326 PMC3528180

[B21] Verwey NA Kester MI van der Flier WM Veerhuis R Berkhof H Twaalfhoven H Blankenstein MA Scheltens AP Pijnenburg YA Additional value of CSF amyloid-beta 40 levels in the differentiation between FTLD and control subjects J Alzheimers Dis 2010 20 445 452 20164558 10.3233/JAD-2010-1392

[B22] Gorno-Tempini ML Hillis AE Weintraub S Kertesz A Mendez M Cappa SF Ogar JM Rohrer JD Black S Boeve BF Classification of primary progressive aphasia and its variants Neurology 2011 76 1006 1014 10.1212/WNL.0b013e31821103e6 21325651 PMC3059138

[B23] Snowden JS Thompson JC Stopford CL Richardson AM Gerhard A Neary D Mann DM The clinical diagnosis of early-onset dementias: diagnostic accuracy and clinicopathological relationships Brain 2011 134 2478 2492 10.1093/brain/awr189 21840888

[B24] Gorno-Tempini ML Brambati SM Ginex V Ogar J Dronkers NF Marcone A Perani D Garibotto V Cappa SF Miller BL The logopenic/phonological variant of primary progressive aphasia Neurology 2008 71 1227 1234 10.1212/01.wnl.0000320506.79811.da 18633132 PMC2676989

[B25] Rabinovici GD Jagust WJ Furst AJ Ogar JM Racine CA Mormino EC O’Neil JP Lal RA Dronkers NF Miller BL Abeta amyloid and glucose metabolism in three variants of primary progressive aphasia Ann Neurol 2008 64 388 401 10.1002/ana.21451 18991338 PMC2648510

[B26] Phukan J Pender NP Hardiman O Cognitive impairment in amyotrophic lateral sclerosis Lancet Neurol 2007 6 994 1003 10.1016/S1474-4422(07)70265-X 17945153

[B27] Ringholz GM Appel SH Bradshaw M Cooke NA Mosnik DM Schulz PE Prevalence and patterns of cognitive impairment in sporadic ALS Neurology 2005 65 586 590 10.1212/01.wnl.0000172911.39167.b6 16116120

[B28] van Langenhove T van der Zee J van Broeckhoven C The molecular basis of the frontotemporal lobar degeneration-amyotrophic lateral sclerosis spectrum Ann Med 2012 44 817 828 10.3109/07853890.2012.665471 22420316 PMC3529157

[B29] Soma K Fu YJ Wakabayashi K Onodera O Kakita A Takahashi H Co-occurrence of argyrophilic grain disease in sporadic amyotrophic lateral sclerosis Neuropathol Appl Neurobiol 2012 38 54 60 10.1111/j.1365-2990.2011.01175.x 21702760

[B30] Spillantini MG Bird TD Ghetti B Frontotemporal dementia and Parkinsonism linked to chromosome 17: a new group of tauopathies Brain Pathol 1998 8 387 402 9546295 10.1111/j.1750-3639.1998.tb00162.x PMC8098460

[B31] Ferrer I Santpere G van Leeuwen FW Argyrophilic grain disease Brain 2008 131 1416 1432 10.1093/brain/awm305 18234698

[B32] Zhang Y Schuff N Du AT Rosen HJ Kramer JH Gorno-Tempini ML Miller BL Weiner MW White matter damage in frontotemporal dementia and Alzheimer’s disease measured by diffusion MRI Brain 2009 132 2579 2592 10.1093/brain/awp071 19439421 PMC2732263

[B33] Varma AR Adams W Lloyd JJ Carson KJ Snowden JS Testa HJ Jackson A Neary D Diagnostic patterns of regional atrophy on MRI and regional cerebral blood flow change on SPECT in young onset patients with Alzheimer’s disease, frontotemporal dementia and vascular dementia Acta Neurol Scand 2002 105 261 269 10.1034/j.1600-0404.2002.1o148.x 11939938

[B34] Kanda T Ishii K Uemura T Miyamoto N Yoshikawa T Kono AK Mori E Comparison of grey matter and metabolic reductions in frontotemporal dementia using FDG-PET and voxel-based morphometric MR studies Eur J Nucl Med Mol Imaging 2008 35 2227 2234 10.1007/s00259-008-0871-5 18661129

[B35] Grossman M The non-fluent/agrammatic variant of primary progressive aphasia Lancet Neurol 2012 11 545 555 10.1016/S1474-4422(12)70099-6 22608668 PMC3361730

[B36] Grossman M Powers J Ash S McMillan C Burkholder L Irwin D Trojanowski JQ Disruption of large-scale neural networks in non-fluent/agrammatic variant primary progressive aphasia associated with frontotemporal degeneration pathology Brain Lang 2012 in Press, doi: 10.1016/j.bandl.2012.10.005. [Epub ahead of print], PMID: 23218686 10.1016/j.bandl.2012.10.005 PMC3610841 23218686

[B37] Hu WT McMillan C Libon D Leight S Forman M Lee VM Trojanowski JQ Grossman M Multimodal predictors for Alzheimer disease in nonfluent primary progressive aphasia Neurology 2010 75 595 602 10.1212/WNL.0b013e3181ed9c52 20713948 PMC2931765

[B38] Engler H Santillo AF Wang SX Lindau M Savitcheva I Nordberg A Lannfelt L Langstrom B Kilander L In vivo amyloid imaging with PET in frontotemporal dementia Eur J Nucl Med Mol Imaging 2008 35 100 106 10.1007/s00259-007-0523-1 17846768

[B39] Rowe CC Ng S Ackermann U Gong SJ Pike K Savage G Cowie TF Dickinson KL Maruff P Darby D Imaging beta-amyloid burden in aging and dementia Neurology 2007 68 1718 1725 10.1212/01.wnl.0000261919.22630.ea 17502554

[B40] Fodero-Tavoletti MT Okamura N Furumoto S Mulligan RS Connor AR McLean CA Cao D Rigopoulos A Cartwright GA O’Keefe G 18 F-THK523: a novel in vivo tau imaging ligand for Alzheimer’s disease Brain 2011 134 1089 1100 10.1093/brain/awr038 21436112

[B41] Broe M Hodges JR Schofield E Shepherd CE Kril JJ Halliday GM Staging disease severity in pathologically confirmed cases of frontotemporal dementia Neurology 2003 60 1005 1011 10.1212/01.WNL.0000052685.09194.39 12654969

[B42] Kersaitis C Halliday GM Kril JJ Regional and cellular pathology in frontotemporal dementia: relationship to stage of disease in cases with and without Pick bodies Acta Neuropathol 2004 108 515 523 10.1007/s00401-004-0917-0 15368070

[B43] Mackenzie IR Rademakers R Neumann M TDP-43 and FUS in amyotrophic lateral sclerosis and frontotemporal dementia Lancet Neurol 2010 9 995 1007 10.1016/S1474-4422(10)70195-2 20864052

[B44] Hoglinger GU Melhem NM Dickson DW Sleiman PM Wang LS Klei L Rademakers R de Silva R Litvan I Riley DE Identification of common variants influencing risk of the tauopathy progressive supranuclear palsy Nat Genet 2011 43 699 705 10.1038/ng.859 21685912 PMC3125476

[B45] Boeve BF The multiple phenotypes of corticobasal syndrome and corticobasal degeneration: implications for further study J Mol Neurosci 2011 45 350 353 10.1007/s12031-011-9624-1 21853287

[B46] Mackenzie IR Neumann M Bigio EH Cairns NJ Alafuzoff I Kril J Kovacs GG Ghetti B Halliday G Holm IE Nomenclature for neuropathologic subtypes of frontotemporal lobar degeneration: consensus recommendations Acta Neuropathol 2009 117 15 18 10.1007/s00401-008-0460-5 19015862 PMC2710877

[B47] Benajiba L Le Ber I Camuzat A Lacoste M Thomas-Anterion C Couratier P Legallic S Salachas F Hannequin D Decousus M TARDBP mutations in motoneuron disease with frontotemporal lobar degeneration Ann Neurol 2009 65 470 473 10.1002/ana.21612 19350673

[B48] Neumann M Sampathu DM Kwong LK Truax AC Micsenyi MC Chou TT Bruce J Schuck T Grossman M Clark CM Ubiquitinated TDP-43 in frontotemporal lobar degeneration and amyotrophic lateral sclerosis Science 2006 314 130 133 10.1126/science.1134108 17023659

[B49] Mackenzie IR Foti D Woulfe J Hurwitz TA Atypical frontotemporal lobar degeneration with ubiquitin-positive, TDP-43-negative neuronal inclusions Brain 2008 131 1282 1293 18362096 10.1093/brain/awn061

[B50] Grossman M Wood EM Moore P Neumann M Kwong L Forman MS Clark CM McCluskey LF Miller BL Lee VM TDP-43 pathologic lesions and clinical phenotype in frontotemporal lobar degeneration with ubiquitin-positive inclusions Arch Neurol 2007 64 1449 1454 10.1001/archneur.64.10.1449 17923628

[B51] Mackenzie IR Munoz DG Kusaka H Yokota O Ishihara K Roeber S Kretzschmar HA Cairns NJ Neumann M Distinct pathological subtypes of FTLD-FUS Acta Neuropathol 2011 121 207 218 10.1007/s00401-010-0764-0 21052700

[B52] Roeber S Mackenzie IR Kretzschmar HA Neumann M TDP-43-negative FTLD-U is a significant new clinico-pathological subtype of FTLD Acta Neuropathol 2008 116 147 157 10.1007/s00401-008-0395-x 18536926

[B53] Rohrer JD Guerreiro R Vandrovcova J Uphill J Reiman D Beck J Isaacs AM Authier A Ferrari R Fox NC The heritability and genetics of frontotemporal lobar degeneration Neurology 2009 73 1451 1456 10.1212/WNL.0b013e3181bf997a 19884572 PMC2779007

[B54] Giannakopoulos P Hof PR Bouras C Dementia lacking distinctive histopathology: clinicopathological evaluation of 32 cases Acta Neuropathol 1995 89 346 355 10.1007/BF00309628 7610766

[B55] Josephs KA Jones AG Dickson DW Hippocampal sclerosis and ubiquitin-positive inclusions in dementia lacking distinctive histopathology Dement Geriatr Cogn Disord 2004 17 342 345 10.1159/000077168 15178950

[B56] Gainotti G Different patterns of famous people recognition disorders in patients with right and left anterior temporal lesions: a systematic review Neuropsychologia 2007 45 1591 1607 10.1016/j.neuropsychologia.2006.12.013 17275042

[B57] Rabinovici GD Miller BL Frontotemporal lobar degeneration: epidemiology, pathophysiology, diagnosis and management CNS Drugs 2010 24 375 398 10.2165/11533100-000000000-00000 20369906 PMC2916644

[B58] Josephs KA Whitwell JL Duffy JR Vanvoorst WA Strand EA Hu WT Boeve BF Graff-Radford NR Parisi JE Knopman DS Progressive aphasia secondary to Alzheimer disease vs FTLD pathology Neurology 2008 70 25 34 10.1212/01.wnl.0000287073.12737.35 18166704 PMC2749307

[B59] Mesulam M Wicklund A Johnson N Rogalski E Leger GC Rademaker A Weintraub S Bigio EH Alzheimer and frontotemporal pathology in subsets of primary progressive aphasia Ann Neurol 2008 63 709 719 10.1002/ana.21388 18412267 PMC2858311

[B60] Rohrer JD Rossor MN Warren JD Alzheimer’s pathology in primary progressive aphasia Neurobiol Aging 2012 33 744 752 10.1016/j.neurobiolaging.2010.05.020 20580129 PMC3314936

[B61] Neary D Snowden J Mann D Frontotemporal dementia Lancet Neurol 2005 4 771 780 10.1016/S1474-4422(05)70223-4 16239184

[B62] Goldman JS Farmer JM Wood EM Johnson JK Boxer A Neuhaus J Lomen-Hoerth C Wilhelmsen KC Lee VM Grossman M Comparison of family histories in FTLD subtypes and related tauopathies Neurology 2005 65 1817 1819 10.1212/01.wnl.0000187068.92184.63 16344531

[B63] Hutton M Lendon CL Rizzu P Baker M Froelich S Houlden H Pickering-Brown S Chakraverty S Isaacs A Grover A Association of missense and 5^**′**^-splice-site mutations in tau with the inherited dementia FTDP-17 Nature 1998 393 702 705 10.1038/31508 9641683

[B64] Watts GD Wymer J Kovach MJ Mehta SG Mumm S Darvish D Pestronk A Whyte MP Kimonis VE Inclusion body myopathy associated with Paget disease of bone and frontotemporal dementia is caused by mutant valosin-containing protein Nat Genet 2004 36 377 381 10.1038/ng1332 15034582

[B65] Dai RM Li CC Valosin-containing protein is a multi-ubiquitin chain-targeting factor required in ubiquitin-proteasome degradation Nat Cell Biol 2001 3 740 744 10.1038/35087056 11483959

[B66] Holm IE Isaacs AM Mackenzie IR Absence of FUS-immunoreactive pathology in frontotemporal dementia linked to chromosome 3 (FTD-3) caused by mutation in the CHMP2B gene Acta Neuropathol 2009 118 719 720 10.1007/s00401-009-0593-1 19844732

[B67] Skibinski G Parkinson NJ Brown JM Chakrabarti L Lloyd SL Hummerich H Nielsen JE Hodges JR Spillantini MG Thusgaard T Mutations in the endosomal ESCRTIII-complex subunit CHMP2B in frontotemporal dementia Nat Genet 2005 37 806 808 10.1038/ng1609 16041373

[B68] Baker M Mackenzie IR Pickering-Brown SM Gass J Rademakers R Lindholm C Snowden J Adamson J Sadovnick AD Rollinson S Mutations in progranulin cause tau-negative frontotemporal dementia linked to chromosome 17 Nature 2006 442 916 919 10.1038/nature05016 16862116

[B69] Gass J Cannon A Mackenzie IR Boeve B Baker M Adamson J Crook R Melquist S Kuntz K Petersen R Mutations in progranulin are a major cause of ubiquitin-positive frontotemporal lobar degeneration Hum Mol Genet 2006 15 2988 3001 10.1093/hmg/ddl241 16950801

[B70] DeJesus-Hernandez M Mackenzie IR Boeve BF Boxer AL Baker M Rutherford NJ Nicholson AM Finch NA Flynn H Adamson J Expanded GGGGCC hexanucleotide repeat in noncoding region of C9ORF72 causes chromosome 9p-linked FTD and ALS Neuron 2011 72 245 256 10.1016/j.neuron.2011.09.011 21944778 PMC3202986

[B71] Renton AE Majounie E Waite A Simon-Sanchez J Rollinson S Gibbs JR Schymick JC Laaksovirta H van Swieten JC Myllykangas L A hexanucleotide repeat expansion in C9ORF72 is the cause of chromosome 9p21-linked ALS-FTD Neuron 2011 72 257 268 10.1016/j.neuron.2011.09.010 21944779 PMC3200438

[B72] Majounie E Renton AE Mok K Dopper EG Waite A Rollinson S Chio A Restagno G Nicolaou N Simon-Sanchez J Frequency of the C9orf72 hexanucleotide repeat expansion in patients with amyotrophic lateral sclerosis and frontotemporal dementia: a cross-sectional study Lancet Neurol 2012 11 323 330 10.1016/S1474-4422(12)70043-1 22406228 PMC3322422

[B73] Gijselinck I Van Langenhove T van der Zee J Sleegers K Philtjens S Kleinberger G Janssens J Bettens K Van Cauwenberghe C Pereson S A C9orf72 promoter repeat expansion in a Flanders-Belgian cohort with disorders of the frontotemporal lobar degeneration-amyotrophic lateral sclerosis spectrum: a gene identification study Lancet Neurol 2012 11 54 65 10.1016/S1474-4422(11)70261-7 22154785

[B74] Whitwell JL Xu J Mandrekar J Boeve BF Knopman DS Parisi JE Senjem ML Dickson DW Petersen RC Rademakers R Frontal asymmetry in behavioral variant frontotemporal dementia: clinicoimaging and pathogenetic correlates Neurobiol Aging 2012 34 636 639 22502999 10.1016/j.neurobiolaging.2012.03.009 PMC3404265

[B75] Arighi A Fumagalli GG Jacini F Fenoglio C Ghezzi L Pietroboni AM De Riz M Serpente M Ridolfi E Bonsi R Early onset behavioral variant frontotemporal dementia due to the C9ORF72 hexanucleotide repeat expansion: psychiatric clinical presentations J Alzheimers Dis 2012 31 447 452 22571983 10.3233/JAD-2012-120523

[B76] Al-Sarraj S King A Troakes C Smith B Maekawa S Bodi I Rogelj B Al-Chalabi A Hortobagyi T Shaw CE p62 positive, TDP-43 negative, neuronal cytoplasmic and intranuclear inclusions in the cerebellum and hippocampus define the pathology of C9orf72-linked FTLD and MND/ALS Acta Neuropathol 2011 122 691 702 10.1007/s00401-011-0911-2 22101323

[B77] Boeve BF Boylan KB Graff-Radford NR DeJesus-Hernandez M Knopman DS Pedraza O Vemuri P Jones D Lowe V Murray ME Characterization of frontotemporal dementia and/or amyotrophic lateral sclerosis associated with the GGGGCC repeat expansion in C9ORF72 Brain 2012 135 765 783 10.1093/brain/aws004 22366793 PMC3286335

[B78] Sieben A Van Langenhove T Engelborghs S Martin JJ Boon P Cras P De Deyn PP Santens P Van Broeckhoven C Cruts M The genetics and neuropathology of frontotemporal lobar degeneration Acta Neuropathol 2012 124 353 372 10.1007/s00401-012-1029-x 22890575 PMC3422616

[B79] Edwards TL Scott WK Almonte C Burt A Powell EH Beecham GW Wang L Zuchner S Konidari I Wang G Genome-wide association study confirms SNPs in SNCA and the MAPT region as common risk factors for Parkinson disease Ann Hum Genet 2010 74 97 109 10.1111/j.1469-1809.2009.00560.x 20070850 PMC2853717

[B80] Rademakers R Cruts M van Broeckhoven C The role of tau (MAPT) in frontotemporal dementia and related tauopathies Hum Mutat 2004 24 277 295 10.1002/humu.20086 15365985

[B81] Mackenzie IR Neumann M Baborie A Sampathu DM Du Plessis D Jaros E Perry RH Trojanowski JQ Mann DM Lee VM A harmonized classification system for FTLD-TDP pathology Acta Neuropathol 2011 122 111 113 10.1007/s00401-011-0845-8 21644037 PMC3285143

[B82] He Z Bateman A Progranulin (granulin-epithelin precursor, PC-cell-derived growth factor, acrogranin) mediates tissue repair and tumorigenesis J Mol Med (Berl) 2003 81 600 612 10.1007/s00109-003-0474-3 12928786

[B83] Van Damme P Van Hoecke A Lambrechts D Vanacker P Bogaert E van Swieten J Carmeliet P Van Den Bosch L Robberecht W Progranulin functions as a neurotrophic factor to regulate neurite outgrowth and enhance neuronal survival J Cell Biol 2008 181 37 41 10.1083/jcb.200712039 18378771 PMC2287280

[B84] Yin F Banerjee R Thomas B Zhou P Qian L Jia T Ma X Ma Y Iadecola C Beal MF Exaggerated inflammation, impaired host defense, and neuropathology in progranulin-deficient mice J Exp Med 2010 207 117 128 10.1084/jem.20091568 20026663 PMC2812536

[B85] Weihl CC Pestronk A Kimonis VE Valosin-containing protein disease: inclusion body myopathy with Paget’s disease of the bone and fronto-temporal dementia Neuromuscul Disord 2009 19 308 315 10.1016/j.nmd.2009.01.009 19380227 PMC2859037

[B86] Ju JS Weihl CC p97/VCP at the intersection of the autophagy and the ubiquitin proteasome system Autophagy 2010 6 283 285 10.4161/auto.6.2.11063 20083896 PMC2997346

[B87] Ju JS Weihl CC Inclusion body myopathy, Paget’s disease of the bone and fronto-temporal dementia: a disorder of autophagy Hum Mol Genet 2010 19 R38 R45 10.1093/hmg/ddq157 20410287 PMC2875057

[B88] Suzuki M Lee HC Kayasuga Y Chiba S Nedachi T Matsuwaki T Yamanouchi K Nishihara M Roles of progranulin in sexual differentiation of the developing brain and adult neurogenesis J Reprod Dev 2009 55 351 355 10.1262/jrd.20249 19721334 PMC2739127

[B89] Ahmed Z Sheng H Xu YF Lin WL Innes AE Gass J Yu X Wuertzer CA Hou H Chiba S Accelerated lipofuscinosis and ubiquitination in granulin knockout mice suggest a role for progranulin in successful aging Am J Pathol 2010 177 311 324 10.2353/ajpath.2010.090915 20522652 PMC2893674

[B90] Gijselinck I Van Broeckhoven C Cruts M Granulin mutations associated with frontotemporal lobar degeneration and related disorders: an update Hum Mutat 2008 29 1373 1386 10.1002/humu.20785 18543312

